# A new approach for simulating inhomogeneous chemical kinetics

**DOI:** 10.1038/s41598-023-39741-y

**Published:** 2023-08-28

**Authors:** Georgia Bradshaw, Mel O’Leary, Arthur Cottrell-Purser, Balder Villagomez-Bernabe, Cyrus Wyett, Frederick Currell, Marcus Webb

**Affiliations:** 1 Department of Mathematics, University of Manchester, Oxford Rd, Manchester, M13 9PL UK; 2 Department of Chemistry, University of Manchester, Oxford Rd, Manchester, M13 9PL UK; 3 Dalton Cumbrian Facility, West Lakes Science and Technology Park, Moor Row, CA24 3HA UK; 4 St Luke’s Cancer Centre, The Royal Hospital, Egerton Rd, Guildford, GU2 7XX UK

**Keywords:** Chemistry, Mathematics and computing

## Abstract

In this paper, inhomogeneous chemical kinetics are simulated by describing the concentrations of interacting chemical species by a linear expansion of basis functions in such a manner that the coupled reaction and diffusion processes are propagated through time efficiently by tailor-made numerical methods. The approach is illustrated through modelling $\alpha$- and $\gamma$-radiolysis in thin layers of water and at their solid interfaces from the start of the chemical phase until equilibrium was established. The method’s efficiency is such that hundreds of such systems can be modelled in a few hours using a single core of a typical laptop, allowing the investigation of the effects of the underlying parameter space. Illustrative calculations showing the effects of changing dose-rate and water-layer thickness are presented. Other simulations are presented which show the approach’s capability to solve problems with spherical symmetry (an approximation to an isolated radiolytic spur), where the hollowing out of an initial Gaussian distribution is observed, in line with previous calculations. These illustrative simulations show the generality and the computational efficiency of this approach to solving reaction-diffusion problems. Furthermore, these example simulations illustrate the method’s suitability for simulating solid-fluid interfaces, which have received a lot of experimental attention in contrast to the lack of computational studies.

## Introduction

The physical universe is composed of two things: radiation and matter. The interaction between them is of fundamental interest to understanding the world around us. An important application of the study of high energy radiation-matter interactions is understanding the processes induced when ionizing radiation interacts with liquids or gasses. Of particular interest are the interactions with water due to its ubiquity, key role in biological systems and ability to produce radiolytic hydrogen.

Ionising radiation interacting with a molecule in fluid can either excite or ionize it, thereby instigating a cascade of processes. These processes are typically divided into three epochs called the physical, physico-chemical and chemical stages^1^. Specifically for water: the physical stage (around $10^{-18}$ s after the initial interaction) consists of energy deposition and fast relaxation leading to the formation of both ionized and excited water molecules; the physico-chemical stage (from $10^{-15}$ to $10^{-12}$ s) consists of processes such as ion-molecule reaction, dissociative relaxation, autoionization of excited states, solvation of electrons, hole diffusion and so on; during the chemical stage (from $10^{-12}$ to $10^{-6}$ s) these species react in the tracks produced by the primary or secondary particles, and diffuse throughout the solution^1^.

These processes have implications in a range of areas such as accurately calculating the biological effect of radiation upon patients undergoing radiation-based therapy^2^, and understanding the risks induced and therefore safety procedures required when storing nuclear materials and waste^3–5^. Water radiolysis occurs in any residual water on radioactive oxide nanopowders and the subsequent reactions produce molecular hydrogen, the production of which must be understood and managed^6–9^. Furthermore, radiolysis is relevant to a wide range of fields, including nuclear corrosion^10^, radiation contamination in streams^11^, medical radioisotope production^12^, physical failure analysis in semiconductors^13^, radiation shielding^14^, and synthesizing bimetallic nanoparticles^15^.

The computational modelling of water radiolysis has been developing as a field since the use of the FACSIMILE computer programme in 1977^16, 17^ which was designed for flow chemistry and general initial value problems. Subsequently, more specialised codes have been developed based on Kinetic Monte Carlo (KMC) methods. These include FLUKA^18^, developed at CERN for work on hadron cascades; PARTRAC^19, 20^, the first code to incorporate chemistry into KMC methods; RITRACKS^21, 22^, developed at NASA to understand the effects of space radiation at the biological level; and those based on Geant4^23, 24^, a general toolkit for simulating the passage of particles through matter. Codes such as the Geant4-DNA extension TOPAS-nBio^25, 26^ have since been developed using the Independent Reaction Time (IRT) model^27–30^ which use KMC methods but treat each chemical reaction independently^31^. This model enables a rapid calculation of radiochemical yields; however, it does not track the spatial distribution of the chemical species as opposed to its step-by-step (SBS)^27^ counterpart and therefore cannot encapsulate the entire behaviour of particles.

KMC methods are well suited to the high energy regime because such systems can be thought of as purely physical, with interactions occurring too quickly for diffusion and chemistry to have an effect. At lower energies however, diffusion and chemical reactions are the drivers of the physico-chemical and chemical stages that the system must undergo to reach equilibrium^1^. Although KMC is adaptable to model the physico-chemical and chemical stages, it is computationally expensive—an inherent limitation well recognised by the community. Efforts are being made to mitigate this expense: Erban and Chapman^32^ propose compartment-based models for tracking molecules, Peukert^33^ stops the primary particle once it deposits a certain amount of energy into the media, and Shin^34^ increases the time step at which reactions are sampled. All of these approaches incur penalties in the completeness of the model, which may lead to a loss of accuracy.

Reaction-diffusion models have been proposed to simulate the physico-chemical and chemical stages of the interactions^24, 26^. Our contention is that spectral methods are well suited to solving reaction-diffusion equations and Debye–Smoluchowski equations prevalant in radiation chemistry and beyond. An alternative class of numerical methods which we do not explore in this paper is the Finite Element Method (FEM)^35^. Spectral methods are extremely efficient at approximating functions that are highly smooth^36^ and diffusive processes tend to have a smoothing effect^37^. The extent to which FEMs can take advantage of smoothness is more limited. The main limitation of spectral methods (particularly when compared with FEM) is that they are restricted to simple domains such as spheres, cuboids and cylinders, but spectral element methods can be used for more complex geometries^38^. In future work we will show that spectral methods have an advantage in efficiently simulating the non-local effects of charge in the Debye–Smoluchowski equation, for which FEM methods generate large dense matrices.

The methodology discussed in this paper is implemented in a forthcoming software package which will be freely available and open source.

## Methods

### The physical stage

A complete radiolysis simulation requires simulation of the physical, physico-chemical and chemical stages. For the physical stage, external software such as the widely used GEANT4^23^ can be used to simulate energy deposition and fast relaxation, outputting point cloud data to be converted to a spatial function of concentration, $[\text {A}]({\textbf{x}},t)$, of each chemical species being simulated. Alternatively, initial conditions may be set manually by the user as a function of the spatial domain.

### The reaction–diffusion model

The physico-chemical and chemical stages are then modelled using a reaction-diffusion system, given by Eq. (1)

1 $$\begin{aligned} \frac{\partial \text {[A]}}{\partial t}({\textbf{x}},t) = D \, \nabla ^2 [\text {A}] ({\textbf{x}},t) + R_{\text {[A]}}(\text {[A]}({\textbf{x}},t),\text {[B]}({\textbf{x}},t),\text {[C]}({\textbf{x}},t)\ldots ), \end{aligned}$$

where D is the diffusion coefficient; $\nabla ^2$ the Laplacian operator; $\text {[A], [B] and [C]}$ denote the concentrations of chemical species $\text { A, B and C}$ respectively, all functions of position ${\textbf{x}}$ and time *t*; and $R_{\text {[A]}}$ is the reaction term associated with species A^39^. In a general chemical kinetic system, similar equations will exist for the chemical species $\text{ B }$, $\text{ C }$, $\ldots$. The concentrations of these different species couple through the chemical network, represented by reaction terms $R_{\text {[A]}}$, $R_{\text {[B]}}$, $R_{\text {[C]}}$, etc.

The use of bold symbols indicates a vector of values, e.g. in general, ${\textbf{x}}$ represents 3 spatial dimensions. This vector notation allows us to collect this system of equations into one equation,

2 $$\begin{aligned} \frac{\partial \boldsymbol{\rho }}{\partial t}({\textbf{x}},t) = {\textbf{D}} \, \nabla ^2 \boldsymbol{\rho } ({\textbf{x}},t) + {\textbf{R}}(\boldsymbol{\rho }({\textbf{x}},t)), \end{aligned}$$

where $\boldsymbol{\rho }({\textbf{x}},t)$ is a vector containing the concentrations of all of the species. The first term in this equation describes the diffusion of all the chemical species through the medium, with ${\textbf{D}}$ containing the diffusion coefficients of the chemical species, and ${\textbf{R}}(\boldsymbol{\rho }({\textbf{x}},t))$ representing the full reaction network.

In one linear spatial dimension, this becomes

3 $$\begin{aligned} \frac{\partial \boldsymbol{\rho }}{\partial t}(x,t) = {\textbf{D}} \, \frac{\partial ^2 \boldsymbol{\rho }}{\partial x^2} (x,t) + {\textbf{R}}(\boldsymbol{\rho }(x,t)). \end{aligned}$$

For spherically symmetric systems, in spherical polar coordinates the reaction-diffusion equation is given by

4 $$\begin{aligned} \frac{\partial \boldsymbol{\rho }}{\partial t}(r,t) = {\textbf{D}} \frac{\partial ^2 (r\boldsymbol{\rho })}{\partial r^2}(r,t) + {\textbf{R}}(\boldsymbol{\rho }(r,t)), \end{aligned}$$

where *r* is the distance from the origin.

### Splitting methods

Using spectral methods, the diffusion equation can be solved analytically for each time step, $t + \Delta t$, giving independent solutions for each spectral coefficient; whereas in spatial value space, the reaction equation can be solved as independent homogeneous chemistry problems at each spatial point, for each chemical species. As these methods eliminate interdependence of solutions between points, it is more efficient to use splitting methods to solve the diffusion and reaction parts of the equation separately and combine them using a stepping technique^40^, rather than using iterative methods for the full partial differential equation (PDE).

**Figure 1:**
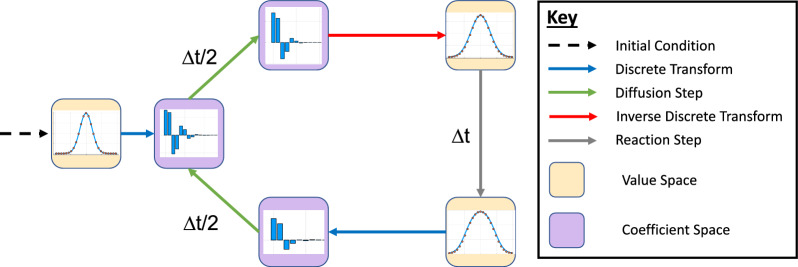
Flow chart showing order of processes for a single iteration of the Strang split-step method^60^. One loop represents a single complete timestep of length $\Delta t$ for the reaction-diffusion equation (two diffusion steps of length $\Delta t/2$ and one reaction step of length $\Delta t$). The graphs in each box are a schematic to represent whether the concentrations are stored as values or coefficients at that point in the process.

The Strang split step method^41^ works by taking a small time step $\Delta t$, and simulating diffusion with the reaction paused for half a time step, then simulating reaction with the diffusion paused for a full time step, then simulating diffusion with reaction paused for a half time step (Fig. 1). The formulation of this method using two half time-steps for diffusion rather than a full time-step incurs an error ${\mathscr {O}}(\Delta t^3)$ per time-step, rather than ${\mathscr {O}}(\Delta t^2)$ per time-step.

These errors are tracked and controlled using an adaptive time step method, where if the error becomes larger than a given tolerance, the time step is decreased and the step is recalculated so that the error always remains below the given tolerance^42^. Furthermore, if the error is significantly lower than the tolerance, the time step is doubled to avoid overcompensation. The error estimate is made using the maximum absolute value of the differences in coefficients $c_n(t)$ in the spectral basis (see below).

### Spectral methods

A spectral method approximates a function, in this case the concentration of a single chemical species $\text{[ }A]$, as a linear combination of $N+1$ basis functions, $\phi ^A_n(x)$, with time-varying spectral coefficients $a_n(t)$^43^:

5 $$\begin{aligned}{}[\text{ A}](x,t)= \rho _\text {A}(x,t) = \sum ^{N}_{n=0} a_n(t) \phi ^\text {A}_n(x) \end{aligned}$$

Each chemical species can be approximated like this, with each species having its own set of spectral coefficients $a_n(t)$, $b_n(t)$, $\ldots$ and its own (possibly distinct) basis functions, $\phi ^\text {A}_n(x)$, $\phi ^\text {B}_n(x)$, $\ldots$.

We can drop the subscripts and superscripts in what follows, denoting a concentration of a species by $\rho (x,t)$ which is expanded in a basis $\phi _n(x)$ with spectral coefficients $c_n(t)$. For each chemical species, the basis function, $\phi _n(x)$, is specially chosen so that it obeys the boundary conditions of the problem domain, meaning that any linear combination of those basis functions will also obey the boundary conditions. Different types of basis functions are suited to different types of domains (e.g. trigonometric functions for an interval domain $\Omega = {\mathbb {I}}$, Hermite-Gaussian functions for an infinite domain $\Omega = {\mathbb {R}}$ and Bessel functions for a disk domain $\Omega = {\mathbb {D}}$)^43, 44^. In this work we look at trigonometric bases for the interval domain $\Omega = [0,L]$ (Table 1).

**Table 1:** Boundary conditions and corresponding basis functions that obey these boundary conditions.

Boundary conditions	Basis function	$\omega _n$
$\rho (0,t) = 0, \quad \frac{\partial \rho }{\partial x}(L,t) = 0$	$\sin (\omega _n x)$	$\frac{\pi }{L}\left( n + \frac{1}{2} \right)$
$\frac{\partial \rho }{\partial x}(0,t) = 0, \quad \rho (L,t) = 0$	$\cos (\omega _n x)$	$\frac{\pi }{L}\left( n + \frac{1}{2} \right)$
$\rho (0,t) = 0, \quad \rho (L,t) = 0$	$\sin (\omega _n x)$	$\frac{\pi }{L}n$
$\frac{\partial \rho }{\partial x}(0,t) = 0, \quad \frac{\partial \rho }{\partial x}(L,t) = 0$	$\cos (\omega _n x)$	$\frac{\pi }{L}n$

$\rho (x,t) = 0$ is the Dirichlet boundary condition^58^ indicating that the concentration of the chemical species is zero at *x* (a perfect sink), and $\frac{\partial \rho }{\partial x}(x,t) = 0$ is the Neumann boundary condition^59^ indicating that the net flux the chemical species is zero at *x*.

For spherically symmetric systems in three dimensions, i.e. Eq. (4), we use a basis that varies only in the distance from the origin *r*, where $a< r < b$ for some nonnegative limits *a* and *b*. The bases we use take the form

6 $$\begin{aligned} \phi _n(r) = \frac{1}{r}\sin (\omega _n (r-a)), \end{aligned}$$

where the frequencies $\omega _n$ depend on the desired boundary conditions. Transforms for these bases utilising the DCT and DST can be designed for some choices of boundary conditions, just as with the trigonometric bases discussed in the main paper.

The following section develops the necessary mathematics for working in a 1D linear domain, i.e. for solving Eq. (3).

### The diffusion step

For the diffusion term

7 $$\begin{aligned} \frac{\partial \boldsymbol{\rho }}{\partial t}(x,t) = D\frac{\partial ^2 \boldsymbol{\rho }}{\partial x^2}(x,t), \end{aligned}$$

the spectral sum, Eq. (5), is substituted into Eq. (7) and solved to give the solution

8 $$\begin{aligned} c_n(t+\Delta t) = c_n(t)\exp (-D \omega ^2_n \Delta t), \end{aligned}$$

where $\omega _n$ for each basis function is given in Table 1. The timestep of each coefficient $c_n$ of each species is independent and therefore only requires ${\mathscr {O}}(N)$ operations for each time step (and is parallelizable). Furthermore, this step is solved *exactly*—there is no truncation error.

### The reaction step

The reaction term,

9 $$\begin{aligned} \frac{\partial \boldsymbol{\rho }}{\partial t}(x,t) = {\textbf{R}}(\boldsymbol{\rho }(x,t)), \end{aligned}$$

is solved by a numerical method. Since the Strang splitting is of second order accuracy, we choose a second order numerical time stepper. Our particular choice is Kahan’s method, because it is an A-stable second order accurate method and only requires the solution of a linear system of equations at each step to achieve this stability (rather than a system of nonlinear equations)^45, 46^. Kahan’s method has also been shown to have advantageous geometrical properties for quadratic vector fields (such as networks of zeroth, first and second order reactions)^47,48^. The time step is as follows.

10 $$\begin{aligned} \boldsymbol{\rho }(x_n,t+\Delta t) \approx \boldsymbol{\rho }(x_n,t) + \left[ {\textbf{I}} - \frac{\Delta t}{2} {{\textbf{J}}}_{{\textbf{R}}}(\boldsymbol{\rho }(x_n,t)) \right] ^{-1}{\textbf{R}}(\boldsymbol{\rho }(x_n,t))\Delta t, \text { for } n = 0,1,\ldots N, \end{aligned}$$

where ${\textbf{I}}$ is the identity matrix, and ${{\textbf{J}}}_{{\textbf{R}}}(\boldsymbol{\rho }(x,t))$ is the Jacobian matrix of the reaction vector ${\textbf{R}}(\boldsymbol{\rho }(x,t))$ with respect to $\rho (x,t)$^49^. One disadvantage of Kahan’s method is that we require the Jacobian matrix, but this is not an imposition because the reaction vector can be written in terms of a (constant) stoichiometric matrix ${\textbf{M}}$ and a reaction rate vector ${\textbf{v}}$, where ${\textbf{R}}(x,t) = {\textbf{M}} \cdot {\textbf{v}}$, with ${\textbf{v}}$ containing only monomials in the species concentrations^50^ (see Supplementary Methods S1 online for example).

The method is applied on the values of the concentrations at the points $x_n$ for $n = 0,1,\ldots , N$, which results in the $N+1$ independent, homogeneous chemical kinetics equations at each point $x_n$. This keeps the operations per timestep at ${\mathscr {O}}(N)$, which is less expensive than performing the timestep on the coefficients (or without splitting at all), which would require simulating a fully coupled system, leading to ${\mathscr {O}}(N^2)$ operations per timestep. The $N+1$ independent steps can also be parallelized.

### Discrete transforms and inverse discrete transforms

The diffusion step acts on spectral coefficients $c_n(t)$ (coefficient space) and the reaction step acts on values $\rho (x_n)$ (value space). Thus we require a method of switching between these two representations, and a choice of $N+1$ points $x_n$ in the spatial domain.

**Figure 2:**
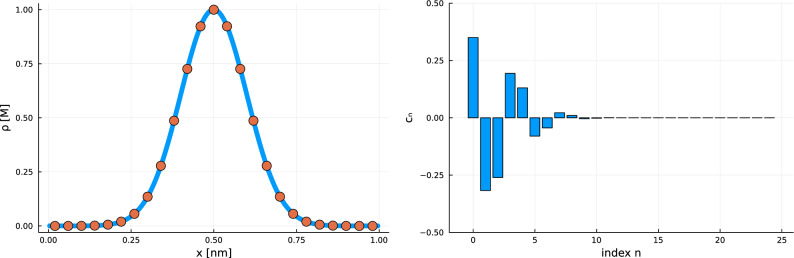
Function $f(x) = \exp \left( -\frac{(x-0.5)^2}{0.02}\right)$ displayed in value space [left] and coefficient space [right]. Left: the function *f*(*x*) and its values on a grid of 25 equally spaced points. Right: the coefficients $c_n$ for $n = 0,1,\ldots ,24$ obtained by taking a discrete transform of the 25 values on the left figure. The right figure demonstrates how only the first few terms of the spectral sum are important, because the coefficients rapidly decay to zero (a consequence of the smoothness of *f*). This means that an accurate approximation can be obtained with relatively few coefficients. Applying an inverse discrete transform on the coefficients in the figure on the right will produce the 25 values of the left figure.

For the bases in Table 1, there are fast transforms that do this, based on versions of the Fast Fourier Transform (FFT)^51^ such as the Discrete Sine Transform (DST) and the Discrete Cosine Transform (DCT) (see Supplementary Methods S1 online for example). These transforms map between values of concentrations at the points $x_n = \left( n+\frac{1}{2} \right) \frac{\pi L}{N+1}$ for $n = 0,1,\ldots N$ and the uniquely determined coefficients $c_n$ for $n = 0,1,\ldots N$. An example of this mapping can be seen in Fig. 2. Furthermore, readily available algorithms can perform DCT and DST in ${\mathscr {O}}(N\log N)$ operations instead of the ${\mathscr {O}}(N^2)$ operations required by a naive approach^51^.

## Results

### Plutonium stewardship

This example explores the effect of radiation incident on thin layers of adsorbed water on surfaces, which applies to the storage and handling of radioactive materials, fuels and wastes^52^. Specifically, effects of $\alpha$-particle irradiation induced radiolysis (produced by radioactive decay of plutonium isotopes in the plutonium oxide powder), and $\gamma$-ray irradiation induced radiolysis (as may be produced by other radionuclides in plutonium oxide powder), are compared. The mechanisms for hydrogen production in these types of systems is not well understood - this can be explored in our software producing fast results allowing for a survey of the role of different parameters. In the simulations presented here, we reduce the problem to a 1D model perpendicular to the plutonium oxide surface by assuming uniform concentration distributions in other directions. We assume that the water-plutonium oxide interface acts as a perfect sink for radiolytically produced chemical species, whilst the water-headspace boundary was treated as a boundary with zero net flux because the rates of transfer of species from water to/from headspace are equal. Hence, a sine basis with Dirichlet-Neumann boundary conditions (Table 1) was used. Other boundary conditions can be investigated in future studies.

In these experiments, the concentrations of $\hbox {H}_2$ at the water-gas boundary were determined at the effective equilibrium state (i.e. where the concentrations of the chemical species are no longer changing significantly over time), for varying thicknesses of water layers, and varying dose rates of incident radiation. This data was gathered using the time step-size $\Delta t = 10^{-3}$ and $N = 100$ spectral terms for water thicknesses of 1-20 monolayers (note that one monolayer of water is approximately 0.25 nm thick). These values were then verified by halving $\Delta t$ and doubling *N*, giving maximum relative differences of 0.00407 Species nm$\vphantom{0}^{-1}$, 0.01106 Species nm$\vphantom{0}^{-1}$ and 0.00772 Species nm$\vphantom{0}^{-1}$ between the two simulations for $\alpha$-radiolysis, $\gamma$-radiolysis using G-values from table 7.4 in Spinks and Woods^53^, and $\gamma$-radiolysis using G-values from Kreipl et al.^20^, respectively. Reaction rate and diffusion coefficients were taken from Kreipl et al.^20^ (see Supplementary Table S2 in Supplementary Data online).

**Figure 3:**
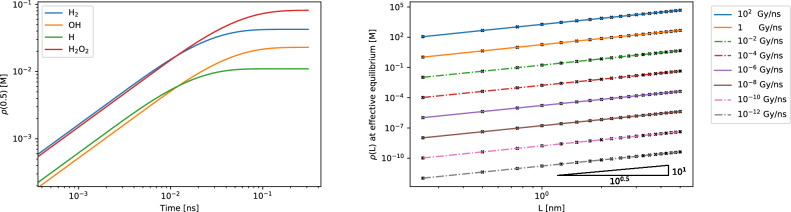
Simulations of incident alpha radiation on thin water layers. Left: a demonstration of the inhomogeneous reaction system reaching equilibrium. The system models incident $\alpha$ radiation with a dose rate of $10^{-2}$ Gy ns$\vphantom{0}^{-1}$ uniformly in a thin film of water of thickness $L = 0.5$ nm, with the dynamics tracked at $x = 0.5$nm (water gas boundary) (see Supplementary Fig. S1 online for sample simulations at varying time steps). Right: results from 160 simulations of incident alpha radiation, with various dose rates, in a thin film of water of different thicknesses ranging from typical dose rates up by over 12 orders of magnitude to illustrate the wide parameter space available. The plotted quantity is the concentration of $\hbox {H}_2$ at the water-gas boundary at equilibrium, as a function of the thickness of water layer for various dose rates. The black crosses show the data points gathered from the simulations, and the gradient triangle in the bottom right verifies that the gradients of the plots show a quadratic dependence between yield of $\hbox {H}_2$ at equilibrium and water thickness $L \in$ [0.25 nm, 5 nm]. G-values^53^ were converted to reaction rate coefficients for the zeroth order reactions used to simulate the constant input of chemical species as a result of irradiation in the physical stage (see Supplementary Table S1 in Supplementary Data online).

**Figure 4:**
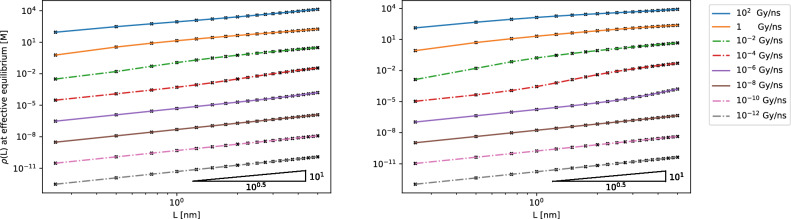
Plots of the concentration of $\hbox {H}_2$ at the water-gas boundary at equilibrium, as a function of the thickness of water layer for various dose rates for Spinks and Woods [left], and Kreipl et al. [right]. The black crosses show the data points gathered from the simulations, and the gradient triangle in the bottom right shows that for gamma radiation, the quadratic dependence between yield of $\hbox {H}_2$ at equilibrium and water thickness *L* does not hold universally. Reaction rate coefficients for the zeroth order reactions used to simulate the constant input of chemical species as a result of irradiation in the physical stage were taken from the G-values from: Table 7.4 in Spinks and Woods^53^ using ‘$\gamma$ and electron radiation with 0.1–20 MeV energies, pH 3–11’ for the left figure, and Table 5 in Kreipl et al.^20^ using the bottom row for the right figure. Diffusion coefficients, reaction network and reaction rates are taken from Tables 2 and 3 in Kreipl et al.^20^, respectively.

Figure 3 (right) shows that for $\alpha$-radiation, the amount of $\hbox {H}_2$ produced has a quadratic dependence on the water layer thickness L, which is suggested by some experimental data^54^. This relationship remains across a wide range of dose rates of $\alpha$-radiation. For $\gamma$-radiation, the nonlinear plots on the logarithmic axis show a more complicated relationship between the amount of $\hbox {H}_2$ produced and water layer thickness L (Fig. 4), requiring more in depth analysis—this behaviour will be the subject of future studies. Furthermore, the difference between the two plots that model $\gamma$-radiation demonstrate the importance of the accuracy of reaction rate coefficients for accurate effective equilibrium results.

### Hollowing out effect

This example demonstrates that the approaches in this paper can adequately simulate the hollowing out effect discussed by Burns et al.^17^. The specific example of the effect explored by Burns et al. is the case where an initial spatial distribution of solvated electrons is wider than that of the hydroxyl radical and the hydrogen ion, in which case the electron concentration can become ‘hollowed out’ over time. While prescribed diffusion methods^55^ fail to simulate this effect, the methods in this paper have no such difficulty. The setup of species and reactions is as in Tables 1 and 3 in Burns et al.^17^, which takes diffusion coefficients and reaction rates from Schwarz^55^ (see Supplementary Table S3 in Supplementary Data).

**Figure 5:**
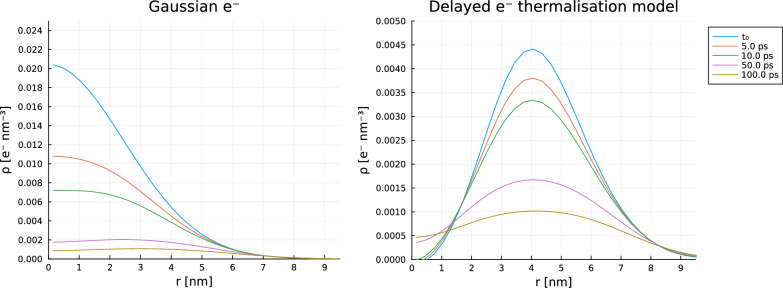
Early spatially inhomogenous time evolution of solvated electrons around an ionising spur. Left: rapid depletion close to the origin due to a fast fourth-order recombination reaction second order in e$\vphantom{0}^{\cdot -}_{aq}$ from a Gaussian initial condition based on Schwarz’ modified prescribed diffusion model, leading to a central minimum forming at 50 ps. Right: distribution with a prescribed central minimum due to a prechemical ballistic transit leading to a slower decay, later proposed by Trumbore^17^. Similar behaviour is seen here using spectral methods as in Burns et al.’s FEM implementation. Simulations were run assuming an ionising spur of energy 100 eV, with 40 coefficients in the radially symmetric basis functions of the form in Eq. (6), over 4606 timesteps. Diffusion coefficients and reaction rate constants were taken from Burns et al.^17^. Simulations were completed in 43 s on a commercial laptop.

**Figure 6:**
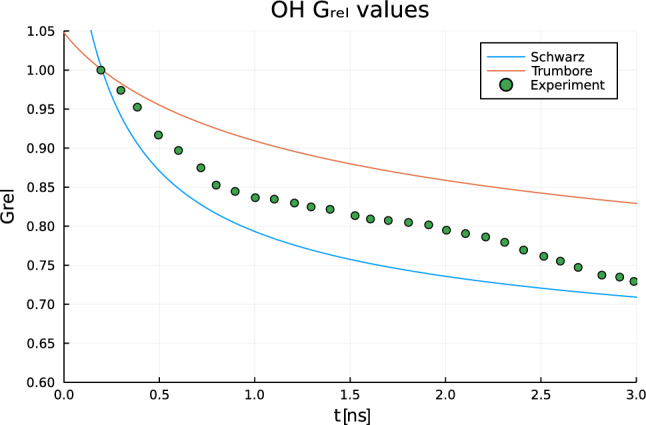
Evolution of relative G of $\vphantom{0}^\cdot$OH, the principle mediator of radiation damage in water radiolysis, from 0-3 ns as a result of initial spatial distributions. Deviation from experiment partly arises due to the breakdown of continuous distributions at the limit of low initial radical numbers per spur noted in^30^ and failure to account for zero-time reaction. Our simulations in the figure above agree with the results of a more intensive but lower resolution FEM implementation of the same models (Ref.^17^, Fig. 21). Parameters used as in Fig. 5.

Early prescribed diffusion models assumed a steady Gaussian distribution of radiolysis products within a spur^56^, but were unable to account for $\hbox {H}_2$ and $\hbox {H}_2$ $\hbox {O}_2$ yields being suppressed in highly pure water and overestimated the prevalence of the cross-combination of HO$\vphantom{0}^\cdot$ with $e^{\cdot -}_{aq}$^55^. The delayed thermalisation-solvation of free electrons liberated during ionisation events results in a hollowed-out initial distribution of solvated electrons, resulting in fewer recombination events and a greater yield of molecular products. Burns et al.^17^ were importantly able to demonstrate numerically that radical recombination reactions of the solvated electron close to the origin of a spherical spur lead to a relative yield profile favouring ionic products over HO$\vphantom{0}^\cdot$. They found the electron distribution rapidly deviates from a Gaussian initial setup, exhibiting a central depression early in their simulations. We show that this behaviour is stably replicated using the approach we present (Fig. 5). The approach we advocate is in keeping with the assessment of these deterministic spur models failing to account for discretisation in the low $N_0$ limit^30^ (Fig. 6).

## Discussion

The simulations presented above demonstrate the potential of spectral methods as a tool to solve reaction-diffusion problems in radiation chemistry. This approach allows for the natural inclusion of boundary conditions, which correspond to chemical reactions at interfaces, or lack thereof (see e.g. Fig. 3). Furthermore, the inherent rapidity of the calculations facilitates performance of many calculations in a short run time, allowing for scanning of the available parameter space to analyse trends in behaviour. For example, the approximate quadratic dependence of the yield of $\hbox {H}_2$ observed across 14 orders of magnitude variation in radiation power, suggests this is a general phenomenon concerning irradiation of thin water layers near a chemically (partially) absorbing boundary. Further discussion of this phenomenon will be the subject of a future paper.

The geometry in the above simulations was restricted to a thin film and a spherically symmetric system, but the diffusion steps and reaction steps generalise naturally to higher dimensions in a way that leverages the existing 1D algorithms.

The work in this paper will be incorporated into a software package that will be open source and freely available. The basic functionality demonstrated in this paper will be taken in several directions.

One direction of current research is to incorporate charge effects explicitly into this software, something which many methodologies lack and instead use artificially inflated reaction rate coefficients as a substitute. The charge effects are being analysed in accordance to the Debye-Smoluchowski equation^27^, where the dynamics of the charge interaction are described by Coulomb’s law with Debye screening^57^. These effects will be resolved in coefficient space using spectral methods, which have an advantage in comparison to the widely used finite element methods for this application due to the non-local effect of charge. This non-local effect results in calculations using dense matrices which become computationally expensive when using FEM, as opposed to calculations using a diagonal matrix when using Spectral Methods. This analysis on the charge effects will be detailed along with examples in a future paper.

Future developments will aim to include Robin boundary conditions (a mixture between a perfect sink and a no-flux boundary), situations where the number of particles in a given region is small (so continuum approximations break down), complex geometries by joining together the simple geometries outlined above, a user-friendly interface and an interface to Monte Carlo codes able to well-describe the physical phase of radiation-matter interactions. This approach to solving the reaction-diffusion equation and Debye–Smoluchowski equation also appears extensible into problems such as electrochemistry and catalysis, another topic for future investigation.

## Conclusion

We have shown the applicability of spectral methods to simulating inhomogeneous chemical kinetics, particularly in the domain of radiation chemistry. Results from two systems have been presented, water-radiolysis involving solid-water interfaces and a model for a single radiolytic spur. The efficiency of the methods developed was illustrated by exploring the effects of dose-rate and water-layer thickness in $\alpha$- and $\gamma$-radiolysis of thin layers of water on absorbing (plutonium oxide) interfaces, a problem which is not amenable to solution using other methods, due to their inability to handle interfaces. Hundreds of results have been presented each of which involved solving the chemical evolution across the entire inhomogeneous chemical phase of the radiolysis, all calculated using a typical laptop. The results are encouraging and suggest the method can be further developed to include the effects of charge, break-down of the continuum representation, systems of higher dimensionality and more complex geometries.

## Supplementary Information

Supplementary Information.

## Data Availability

The datasets used in this paper can be made available upon request to the corresponding author.
