# Supplementary material for: A new approach for simulating inhomogeneous chemical kinetics
Source: Sci Rep. 2023 Aug 28;13:14010. doi: 10.1038/s41598-023-39741-y (PMC10462703; doi:10.1038/s41598-023-39741-y)
Supplement: Supplementary file 1 — Supplementary Information. [file 41598_2023_39741_MOESM1_ESM.pdf]

Supplementary Information: A New Approach for Simulating Inhomogeneous Chemical Kinetics

Georgia Bradshaw[1,*], Mel O'Leary[2,3], Arthur S. F. Purser[2], Balder Villagomez-Bernabe[2,3,4], Cyrus Wyett[2,3], Frederick Currell[2,3], and Marcus Webb[1]

[1] Department of Mathematics, University of Manchester, Oxford Rd, Manchester, M13 9PL, UK
[2] Department of Chemistry, University of Manchester, Oxford Rd, Manchester, M13 9PL, UK
[3] Dalton Cumbrian Facility, West Lakes Science and Technology Park, Moor Row, CA24 3HA, UK
[4] St Luke's Cancer Centre, The Royal Hospital, Egerton Rd, Guildford, GU2 7XX, UK
[*] Georgia.Bradshaw@manchester.ac.uk

Supplementary Methods

Stoichiometry

Given a reaction-diffusion system with the reaction set

Reaction
A + A → B
A + B → C

the system of PDEs is given as

$$\frac{\partial \rho_A}{\partial t}(x,t) = D_A \nabla^2 \rho_A(x,t) - 2\rho_A^2(x,t)k_{AA}^B - \rho_A(x,t)\rho_B(x,t)k_{AB}^C \tag{S1a}$$

$$\frac{\partial \rho_B}{\partial t}(x,t) = D_B \nabla^2 \rho_B(x,t) + 2\rho_A^2(x,t)k_{AA}^B - \rho_A(x,t)\rho_B(x,t)k_{AB}^C \tag{S1b}$$

$$\frac{\partial \rho_C}{\partial t}(x,t) = D_C \nabla^2 \rho_C(x,t) + \rho_A(x,t)\rho_B(x,t)k_{AB}^C, \tag{S1c}$$

where $\rho(x,t)$ is the concentration of the the chemical species given as the subscript, k is the reaction rate coefficient with reactants given as the subscript, and products given as the superscripts. The first term in each of the PDEs describes the diffusion of that species through the medium, and $\mathbf{R}(\rho(x,t))$ can be written in the form

$$\frac{\partial \rho}{\partial t}(x,t) = \mathbf{R}(\rho(x,t)) = \begin{bmatrix} -2\rho_A^2(x,t)k_{AA}^B - \rho_A(x,t)\rho_B(x,t)k_{AB}^C \\ 2\rho_A^2(x,t)k_{AA}^B - \rho_A(x,t)\rho_B(x,t)k_{AB}^C \\ \rho_A(x,t)\rho_B(x,t)k_{AB}^C \end{bmatrix}. \tag{S2}$$

We can now form a stoichiometric matrix, $\mathbf{M}$, and reaction vector, $\mathbf{v}$,

$$\mathbf{M} = \begin{bmatrix} -2 & -1 \\ 2 & -1 \\ 0 & 1 \end{bmatrix}, \tag{S3a}$$

$$\mathbf{v} = \begin{bmatrix} \rho_A^2(x,t)k_{AA}^B \\ \rho_A(x,t)\rho_B(x,t)k_{AB}^C \end{bmatrix}, \tag{S3b}$$

where each column in $\mathbf{M}$ represents a reaction, and each row represents a single chemical species. Each element in $\mathbf{M}$ is denoted by $\pm n$, where n is the number of the product $(+)$ or reactant $(-)$ present in the reaction. Each row in $\mathbf{v}$ represents the reaction rate constants and species for each reaction. Now the reaction vector $\mathbf{R}(\rho(x,t))$ can be represented as $\mathbf{M} \cdot \mathbf{v}$.

The Jacobian matrix, $\mathbf{J_R}(\rho(x,t))$, required for the calculation of a single time step using Kahan's method can now be rewritten as $\mathbf{M}\cdot\mathbf{J_v}(\rho(x,t))$, where $\mathbf{J_v}(\rho(x,t))_{i,j} = \frac{\partial v_i}{\partial \rho(x,t)_j}$. For this example, $\mathbf{J_v}(\rho(x,t))$ is given by

$$\begin{aligned}\mathbf{J_v}(\rho(x,t)) &= \begin{bmatrix} \frac{\partial}{\partial \rho_A(x,t)}(\rho_A^2(x,t)k_{AA}^B) & \frac{\partial}{\partial \rho_B(x,t)}(\rho_A^2(x,t)k_{AA}^B) & \frac{\partial}{\partial \rho_C(x,t)}(\rho_A^2(x,t)k_{AA}^B) \\ \frac{\partial}{\partial \rho_A(x,t)}(\rho_A(x,t)\rho_B(x,t)k_{AB}^C) & \frac{\partial}{\partial \rho_B(x,t)}(\rho_A(x,t)\rho_B(x,t)k_{AB}^C) & \frac{\partial}{\partial \rho_C(x,t)}(\rho_A(x,t)\rho_B(x,t)k_{AB}^C) \end{bmatrix} \\ &= \begin{bmatrix} 2\rho_A(x,t)k_{AA}^B & 0 & 0 \\ \rho_B(x,t)k_{AB}^C & \rho_A(x,t)k_{AB}^C & 0 \end{bmatrix}\end{aligned} \tag{S4}$$

Discrete Transforms

Discrete transforms are used to transform from coefficient space to value space, whilst inverse discrete transforms are used to transform from value space to coefficient space. Given the concentration distribution for an arbitrary basis

$$\rho(x,t) = \sum_{n=0}^{N} c_n(t)\phi(x), \tag{S5}$$

for N distinct spatial values $x_0, x_1, ..., x_N$, this can be rewritten in the form of a matrix multiplication

$$\begin{pmatrix} \rho(x_0) \\ \rho(x_1) \\ .. \\ .. \\ \rho(x_N) \end{pmatrix} = \Phi \cdot \begin{pmatrix} c_0(t) \\ c_1(t) \\ .. \\ .. \\ c_N(t) \end{pmatrix}, \tag{S6}$$

where

$$\Phi = \begin{pmatrix} \phi_0(x_0) & \phi_1(x_0) & .. & .. & \phi_N(x_0) \\ \phi_0(x_1) & \phi_1(x_1) & .. & .. & \phi_N(x_1) \\ .. & .. & .. & .. & .. \\ .. & .. & .. & .. & .. \\ \phi_0(x_N) & \phi_1(x_N) & .. & .. & \phi_N(x_2) \end{pmatrix}. \tag{S7}$$

Given a basis $\{\phi_n\}_{n=0}^N$, the transform can be made fast if we chose points $\{x_k\}_{k=0}^N$ in which the inverse of Φ can be calculated without the need for linear algebra. Below is an example using the trigonometric basis with Dirichlet boundary conditions at $x = 0$ and Neumann boundary conditions at $x = L$, (SinDN basis),

$$\phi(x) = \sin\left(\frac{\pi}{L}\left(n + \frac{1}{2}\right)x\right). \tag{S8}$$

All spatial points in our software package are defined as $x_k = \frac{L(k+\frac{1}{2})}{N+1}$. Choosing N = 1 for simplicity, (S7) becomes

$$\Phi = \begin{pmatrix} \sin(\frac{\pi}{8}) & \sin(\frac{3\pi}{8}) \\ \sin(\frac{3\pi}{8}) & \sin(\frac{9\pi}{8}) \end{pmatrix}, \tag{S9}$$

where $\sin(\frac{9\pi}{8}) = -\sin(\frac{\pi}{8})$. The inverse, Φ^{-1}, can be found as

$$\Phi^{-1} = \begin{pmatrix} -\frac{\sin(\frac{\pi}{8})}{-\sin^2(\frac{\pi}{8})-\cos^2(\frac{\pi}{8})} & -\frac{\cos(\frac{3\pi}{8})}{-\sin^2(\frac{\pi}{8})-\cos^2(\frac{\pi}{8})} \\ -\frac{\cos(\frac{3\pi}{8})}{-\sin^2(\frac{\pi}{8})-\cos^2(\frac{\pi}{8})} & \frac{\sin(\frac{\pi}{8})}{-\sin^2(\frac{\pi}{8})-\cos^2(\frac{\pi}{8})} \end{pmatrix}. \tag{S10}$$

This can be rewritten as

$$\Phi^{-1} = -\frac{1}{\sin^2\left(\frac{\pi}{8}\right) - \cos^2\left(\frac{\pi}{8}\right)} \begin{pmatrix} \sin(\frac{\pi}{8}) & \sin(\frac{3\pi}{8}) \\ \sin(\frac{3\pi}{8}) & -\sin(\frac{\pi}{8}) \end{pmatrix} = -\frac{1}{\sin^2\left(\frac{\pi}{8}\right) - \cos^2\left(\frac{\pi}{8}\right)}\Phi, \tag{S11}$$

Furthermore, it is noted that

$$\mathrm{Det}(\Phi) = \sin^2\left(\frac{\pi}{8}\right) - \sin^2\left(\frac{\pi}{8}\right) \tag{S12}$$

showing that for this example $\Phi^{-1} = \frac{1}{\text{Det}(\Phi)}\Phi$.

Readily available algorithms can perform DCT and DST in $\mathscr{O}(N\log N)$ operations instead of the $\mathscr{O}(N^2)$ operations required by a naive approach[1].

For implementation in our software, we choose the discrete transform best suited to our basis functions. Given the concentration distribution using the SinDN basis

$$\rho(x,t) = \sum_{n=0}^{N} c_n(t)\sin\left(\frac{\pi}{L}\left(n+\frac{1}{2}\right)x\right). \tag{S13}$$

and the spatial points $x_k = \frac{L(k+\frac{1}{2})}{N}$, we can rewrite (S13) as

$$\rho_k(t) = \sum_{n=0}^{N} c_n(t)\sin\left(\pi\frac{\left(n+\frac{1}{2}\right)\left(k+\frac{1}{2}\right)}{N}\right), \tag{S14}$$

where subscript k refers to the kth point in the spatial domain.

We can now compare (S14) to the library of discrete sine transforms[2], and see that it takes the form of the Type-IV Discrete Sine Transform (DST-IV)

$$\rho_k(t) = 2\sum_{n=0}^{N} c_n(t)\sin\left(\pi\frac{(n+\frac{1}{2})(k+\frac{1}{2})}{N}\right), \tag{S15}$$

and so we can express the transform from coefficient space to value space as

$$\{\rho_k(t)\}_{k=0}^{N} = \frac{1}{2}(DSTIV(\{c_n(t)\}_{n=0}^{N})). \tag{S16}$$

To transform from value space to coefficient space, the inverse of the function given in (S16) must be found. As DST-IV is the inverse of itself[2], this is simply given by

$$\{c_n(t)\}_{n=0}^{N} = 2(DSTIV(\{\rho_k(t)\}_{k=0}^{N})). \tag{S17}$$

Analogous processes need to be applied for each basis.

Supplementary Figure S1

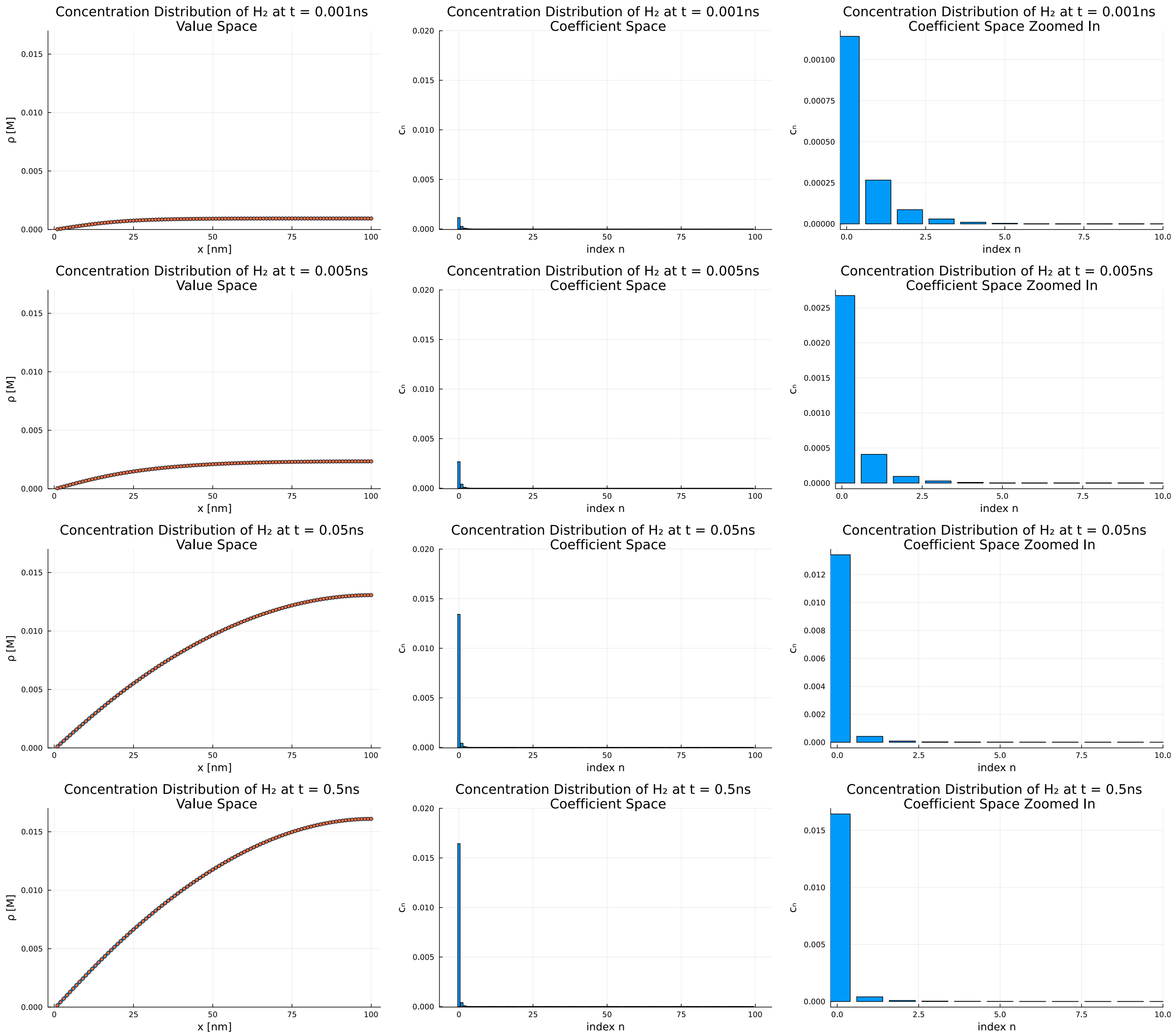

Figure S1. Figure displaying a sample of the simulations ran to produce Left panel of Fig. 3. These plots show the concentration distributions of H_2 for varying points in time, in both value space [Left hand column] and coefficient space [Middle column for a fixed scale, and Right column is zoomed in to show the behaviour of the first 10 coefficients]. As expected, the coefficient representations of these data show that as time progresses, the lowest order term (i.e index 0) becomes more dominant - this is the result of the smoothing effect of the diffusion term.

Supplementary Data

Plutonium Stewardship

In our simulations, radiolytic production was treated as a zero-order chemical reaction. This treatment is equivalent to assuming in the simulated system, that the incident radiation power and medium species' concentration are constant, for the period simulated. This assumption holds on short enough timescales such that the amount of medium species consumed has negligible impact on the medium's overall concentration. When this assumption fails, we would explicitly include the medium species consumption in the reaction network and the radiolytic production as a first order chemical reaction. This explicit model still assumes that the incident radiation power is constant. For a given incident radiation field then the fixed dose rate into the assumed fixed medium is given in Gy s^{-1}. This dose rate was multiplied by the radiolytic yield, in nanomoles per Joule, and the medium's density, kilograms per litre, to yield a rate of production, which was taken in units of Molarity per nanosecond [Table S1]. Note that for the G-values gathered from Kreipl et al[3], 1 Species / 100 eV is equivalent to 103.65655 nmol J^{-1}.

Alpha Radiation		
Chemical Species	G-value from Spinks and Woods[4] [Gy s^{-1}]	Reaction Rate Coefficient [M ns^{-1}]
H_2	0.163	163 $\times$ Dose rate
$H^\bullet$	0.062	62.0 $\times$ Dose rate
H_2O_2	0.150	150 $\times$ Dose rate
$^\bullet OH$	0.052	52.0 $\times$ Dose rate

Gamma Radiation				
Chemical Species	Spinks and Woods[4]		Kreipl et al[3]	
	G-value [Gy s^{-1}]	Reaction Rate Coefficient [M ns^{-1}]	G-value [Species / 100eV]	Reaction Rate Coefficient [M ns^{-1}]
H_2	0.047	47.0 $\times$ Dose Rate	0.16	16.577 $\times$ Dose Rate
$H^\bullet$	0.062	62.0 $\times$ Dose Rate	0.63	65.273 $\times$ Dose Rate
H_2O_2	0.073	73.0 $\times$ Dose Rate	-	-
$^\bullet OH$	0.28	280 $\times$ Dose Rate	5.78	598.852 $\times$ Dose Rate
e^-_{aq}	0.28	280 $\times$ Dose Rate	4.83	500.424 $\times$ Dose Rate
H_3O^+	-	280 $\times$ Dose Rate	4.83	500.424 $\times$ Dose Rate

Table S1. Table collecting all data used to simulate incident radiation as zeroth order reactions. Note that the H_3O^+ G-value missing from Spinks and Woods is assumed to be the same as the e^-_{aq} G-value due to comparison with Kreipl et al.

Table S2 provides all other information required to recreate these simulations .

Chemical Species	Diffusion Coefficient from Kreipl et al[3] [$\times 10^9$ m^2 s^{-1}]	[nm^2 ns^{-1}]
e^-_{aq}	4.9	4.9
$^\bullet OH$	2.8	2.8
$H^\bullet$	7.0	7.0
H_3O^+	9.0	9.0
H_2	4.8	4.8
OH^-	5.0	5.0
H_2O_2	2.3	2.3

Chemical Reaction	Reaction Rate Coefficient from Kreipl et al[3] [M s^{-1}]	[M ns^{-1}]
$e^-_{aq} + e^-_{aq} + 2H_2O \rightarrow H_2 + 2OH^-$	0.5×10^{10}	5.0
$e^-_{aq} + {}^\bullet OH \rightarrow OH^-$	2.95×10^{10}	29.5
$e^-_{aq} + H^\bullet + H_2O \rightarrow H_2 + OH^-$	2.65×10^{10}	26.5
$e^-_{aq} + H_3O^+ \rightarrow H^\bullet + H_2O$	2.11×10^{10}	21.1
$e^-_{aq} + H_2O_2 \rightarrow OH^- + {}^\bullet OH$	1.41×10^{10}	14.1
${}^\bullet OH + {}^\bullet OH \rightarrow H_2O_2$	0.44×10^{10}	4.40
${}^\bullet OH + H^\bullet \rightarrow H_2O$	1.44×10^{10}	14.4
$H^\bullet + H^\bullet \rightarrow H_2$	1.20×10^{10}	12.0
$H_3O^+ + OH^- \rightarrow 2H_2O$	1.43×10^{11}	143

Table S2. Diffusion coefficients and reaction rate coefficients used for all simulations in the plutonium stewardship section. These simulations all use step size $\Delta t = 10^-3$, $N = 100$ spectral terms for water thicknesses, L, of 1-20 monolayers where 1 monolayer is assumed to have thickness of 0.25nm. Initial distributions for all species were set to $\rho(x) = 0$.

Hollowing out Effect

Table S3 provides all information required to recreate the hollowing out effect simulations.

Chemical Species	r_0 from Burns et al[5] [nm]		G_0 from Burns et al[5] [Species / 100eV]	
	Schwarz	Trumbore	Schwarz	Trumbore
e^-_{aq}	2.458	2.309	4.78	4.70
H^+	1.145	2.121	4.78	4.70
H	1.145	2.121	0.62	0.80
OH	1.145	2.121	5.70	6.00

Chemical Species	Initial Distributions from Burns et al[5]	
	Schwarz	Trumbore
e^-_{aq}	$\rho_0(r,t) = \frac{G_0}{(\pi b^2)^{3/2}} \exp(-\frac{r^2}{b^2})$	$\rho_0(r,t) = G_0 \exp(-\frac{r^2}{2r_0^2}) \frac{r^3}{32\pi r_0^6}$
H^+	$\rho_0(r,t) = \frac{G_0}{(\pi b^2)^{3/2}} \exp(-\frac{r^2}{b^2})$	$\rho_0(r,t) = \frac{G_0}{(\pi b^2)^{3/2}} \exp(-\frac{r^2}{b^2})$
H	$\rho_0(r,t) = \frac{G_0}{(\pi b^2)^{3/2}} \exp(-\frac{r^2}{b^2})$	$\rho_0(r,t) = \frac{G_0}{(\pi b^2)^{3/2}} \exp(-\frac{r^2}{b^2})$
OH	$\rho_0(r,t) = \frac{G_0}{(\pi b^2)^{3/2}} \exp(-\frac{r^2}{b^2})$	$\rho_0(r,t) = \frac{G_0}{(\pi b^2)^{3/2}} \exp(-\frac{r^2}{b^2})$

Chemical Species	Diffusion Coefficient from Burns et al[5] [$\times 10^7$ dm^2 s^{-1}]	
	Schwarz	Trumbore
e^-_{aq}	4.5	4.5
H^+	9.0	10.0
H	7.0	8.0
OH	2.8	2.0
OH^-	5.0	2.0
H_2O_2	2.2	1.4

Chemical Reaction	Reaction Rate Coefficient from Burns et al[5] [dm^3 mol^{-1} s^{-1}]	
	Schwarz	Trumbore
$2H_2O + e^-_{aq} + e^-_{aq} \rightarrow H_2 + 2OH^-$	0.55	0.50
$H + H_2O + e^-_{aq} \rightarrow H_2 + OH^-$	2.5	3.0
$H + H \rightarrow H_2$	1.0	1.3
$e^-_{aq} + H^+ \rightarrow H$	1.7	2.3
$e^-_{aq} + OH \rightarrow OH^-$	2.5	3.0
$e^-_{aq} + H_2O_2 \rightarrow OH^- + OH$	1.3	1.23
$H + H_2O_2 \rightarrow H_2O + OH$	0.01	0.016
$H^+ + OH^- \rightarrow H_2O$	10.0	14.3
$OH + OH \rightarrow H_2O_2$	0.6	0.5
$H + OH \rightarrow H_2O$	2.0	3.2
e^-_{aq} + Scavenger$_1 \rightarrow P_1$	0.87	0.87
H + Scavenger$_2 \rightarrow P_2$	0.05	0.05
OH + Scavenger$_3 \rightarrow P_3$	0.13	0.13

Table S3. This table contains all parameters gathered from Burns et al[5] to run the hollowing out effect simulations. The first two panels give information on the initial conditions used, where $b^2 = 2r_0^2$ at $t = 0$. The bottom two panels provide the diffusion coefficients and reaction rate coefficients required to simulate the system. Our simulations used an adaptive time stepping algorithm with 4606 timesteps, N = 40 spectral terms and a spatial domain of r = [0,100] nm.

References

1. Frigo, M. & Johnson, S. The design and implementation of FFTW3. *Proc. IEEE* **93**, 216–231, DOI: https://doi.org/10.1109/JPROC.2004.840301 (2005).

2. Oppenheim, A. V. *Discrete-time signal processing* (Pearson Education India, 1999).

3. Kreipl, M. S., Friedland, W. & Paretzke, H. G. Time-and space-resolved Monte Carlo study of water radiolysis for photon, electron and ion irradiation. *Radiat. environmental biophysics* **48**, 11–20, DOI: https://doi.org/10.1007/s00411-008-0194-8 (2009).

4. Spinks, J. W. T. & Woods, R. J. *An introduction to radiation chemistry* (New York, NY (USA); John Wiley and Sons Inc., 1990).

5. Burns, W. G., Sims, H. E. & Goodall, J. A. B. Radiation chemical diffusion kinetic calculations with prescribed and non-prescribed diffusion—I: Spherical and cylindrical cases. *Radiat. Phys. Chem. (1977)* **23**, 143–180, DOI: https://doi.org/10.1016/0146-5724(84)90105-5 (1984).
